# Inhibition of CK2α down-regulates Notch1 signalling in lung cancer cells

**DOI:** 10.1111/jcmm.12068

**Published:** 2013-05-08

**Authors:** Shulin Zhang, Hao Long, Yi-Lin Yang, Yucheng Wang, David Hsieh, Weiming Li, Alfred Au, Hubert J Stoppler, Zhidong Xu, David M Jablons, Liang You

**Affiliations:** a Thoracic Oncology Laboratory, Department of Surgery, Helen Diller Family Comprehensive Cancer Center, University of California San FranciscoSan Francisco, CA, USA; b Department of Surgical Oncology, The Third Affiliated Hospital of Guangzhou Medical UniversityGuangzhou, China; c Lung Cancer Institute, Sun Yat-sen UniversityGuangzhou, China; d Department of Surgery, Helen Diller Family Comprehensive Cancer Center, University of California San FranciscoSan Francisco, CA, USA; e Tissue Core, Comprehensive Cancer Center, University of California San FranciscoSan Francisco, CA, USA

**Keywords:** CK2α, Notch1, CD44 + /CD24−, cancer stem cell, lung cancer

## Abstract

Protein kinase CK2 is frequently elevated in a variety of human cancers. The Notch1 signalling pathway has been implicated in stem cell maintenance and its aberrant activation has been shown in several types of cancer including lung cancer. Here, we show, for the first time, that CK2α is a positive regulator of Notch1 signalling in lung cancer cell lines A549 and H1299. We found that Notch1 protein level was reduced after CK2α silencing. Down-regulation of Notch1 transcriptional activity was demonstrated after the silencing of CK2α in lung cancer cells. Furthermore, small-molecule CK2α inhibitor CX-4945 led to a dose-dependent inhibition of Notch1 transcriptional activity. Conversely, forced overexpression of CK2α resulted in an increase in Notch1 transcriptional activity. Finally, the inhibition of CK2α led to a reduced proportion of stem-like CD44 + /CD24− cell population. Thus, we report that the inhibition of CK2α down-regulates Notch1 signalling and subsequently reduces a cancer stem-like cell population in human lung cancer cells. Our data suggest that CK2α inhibitors may be beneficial to the lung cancer patients with activated Notch1 signalling.

## Introduction

Protein kinase CK2 (also known as casein kinase II) is a highly conserved serine/threonine kinase that phosphorylates hundreds of proteins 1. CK2α (catalytic subunit of protein kinase CK2) is overexpressed in many human cancers, including lung cancer, and recently emerged as a novel cancer therapeutic target 2, 3. The level of CK2α expression is well regulated in normal cells 4, and the increase in CK2α protein level and activity has been consistently observed in a variety of human cancers 5–7. For instance, the overexpression and/or nuclear localization of CK2α are poor prognosis markers for several human cancers, including acute myeloid leukaemia, chronic lymphocytic leukaemia, prostate cancer and gastric cancer 8–11. CK2α is involved in cell proliferation and survival 12–14, and in several key signalling pathways such as PI3K, NFkB and Wnt 3, 4, 15. Recently, we have shown that CK2α inhibition down-regulates Hedgehog/Gli1 signalling in lung cancer cells 16.

Human Notch is a heterodimeric type I transmembrane receptor protein encoded by one of four Notch genes (Notch1–Notch4). These single-pass transmembrane proteins interact with ligands of the Delta and/or Jagged/Serrate family. The ligands are also transmembrane proteins, which bind to Notch receptors displayed on adjacent cells. Once ligand-receptor binding occurs, the Notch receptor undergoes a conformational change to expose a previously protected site to proteolytic cleavage by metalloprotease and gama-secretase, releasing an extracellular and intracellular fragment, respectively. These catalytic steps cleave the intracellular domain (ICN) into the nucleus, where it can switch the transcription factor CSL from a transcriptional repressor to a transcriptional activator by replacing a corepressor complex with a coactivator complex. The CSL/ICN/coactivator complex transactivates various target genes, including those of the Hes/Hey families. Studies in vertebrate systems suggest that the Notch signalling pathway is highly conserved from drosophila to vertebrates 17. The Notch signalling pathway functions in cell-fate determination and differentiation. A role for Notch signalling in cancer was first suspected with the characterization of t(7;9)(q34;q34.3) chromosomal translocations in a subset of human T cell acute lymphoblastic leukaemia 18. Only 10% of human non–small-cell lung cancer (NSCLC) showed the activating alterations in Notch1 19. An additional 30% of NSCLCs have lost expression of Numb, a negative regulator of Notch, resulting in increased Notch activity 19.

Notch transmembrane receptors (Notch 1–4) are expressed in stem cells and early progenitor cells. Direct evidence that Notch signalling contributes to maintenance of cancer stem cell phenotype derives from a series of studies in different tumour types. For example, Notch1 inhibition alters CD44^+^/CD24^−^ population and reduces the formation of brain metastases from breast cancer 20.

To date, there is no evidence that CK2α is a positive regulator of Notch1 signalling. In this study, we investigated the possible relationship between CK2α and Notch1 signalling in lung cancer cells.

## Materials and methods

### Cell culture and small molecule treatment

Human NSCLC cell lines (A549, A427, H460, H1299, H1650, H358, H838 and H322) were obtained from American Type Culture Collections (Manassas, VA, USA). Cells were routinely maintained in RPMI-1640 supplemented with 10% heat-inactivated foetal bovine serum, penicillin (100 μg/ml) and streptomycin (100 μg/ml). All cells were routinely cultivated at 37°C in a humid incubator with 5% CO_2_. Treatment with CX-4945 (Synkinase, San Diego, CA, USA) and TBB (Sigma-Aldrich, St. Louis, MO, USA) dissolved in dimethyl sulfoxide (DMSO) was administered at several dosages (1, 5 and 10 μM of CX4945; 10 μM of TBB). Cells were grown in medium for 48 hrs after treatment. Cell proliferation *in vitro* was assessed using a CellTiter-Glo Luminescent cell viability assay (Promega Corporation, Madison, WI, USA), according to the manufacturer's protocol 21.

### Tissue samples and immunohistochemistry

Fresh lung cancer tissues were obtained from patients with lung cancer who were undergoing surgical resection of the primary tumour. All human tissue samples were obtained and analysed in accordance with procedures approved by the institutional review board of the University of California, San Francisco (IRB H8714-22 942-01). We obtained written informed consents from all participants involved in our study. The tissue microarray sections were immunostained as previously described 21. Anti-Notch1 antibody was from Cell Signalling (Beverly, MA, USA; D1E11). The following scoring system was employed: −, no stain; +, weak staining (30% or above stained cellularity considered as positive); ++, moderate staining (10% or above stained cellularity considered as positive); +++, strong staining (positive). All scoring systems were under low magnification (10 ×).

### siRNA and plasmid DNA transfection

CK2α siRNA (ON-TARGET *plus* SMARTpool) and control siRNA were purchased from Thermo Scientific (Waltham, MA, USA). In brief, cells were seeded in a 6-well plate as 10^5^ cells/well 1 day before transfection, with a target of 30–50% confluency at the time of transfection. Cells were transfected with 50 nmol/l of siRNA using Lipofectamine RNAiMAX (Invitrogen, Carlsbad, CA, USA) according to the manufacturer's protocol. Adequate inhibition of the siRNA-mediated knockdown was confirmed by Western blot. The pcDNA3.1-CK2α or control pcDNA3.1-LacZ plasmid vectors were then transfected into the A549 cells (0.5 μg/ml in 24-well plate) using Lipofectamine 2000 transfection reagent (Invitrogen), according to the manufacturer's protocol. Cells were harvested for RT-PCR and Western blot or used in reporter assays at 48 hrs post-transfection.

### RNA isolation, cDNA synthesis and semi-quantitative RT-PCR

Isolation of RNA was performed using RNeasy Mini kit (Qiagen, Valencia, CA, USA). Normal human lung total RNA was purchased from Clontech Laboratories (Cat. #: 636524, Mountain View, CA, USA). The normal lung sample was pooled from three Caucasians without lung cancer (aged from 32 to 61). Five-hundred nanogram of total RNA was converted into 20 μl cDNA using iScript cDNA Synthesis Kits (Bio-Rad, Hercules, CA, USA) according to the manufacturer's recommendations. PCR bands were visualized under UV light and photographed.

### Real-time-PCR

A total of 2 μl of the reverse transcription reaction mixture were used as template for real-time detection using TaqMan Technology on an Applied Biosystems 7000 sequence detection system (Applied Biosystems, Foster City, CA, USA). Gene expression was quantified for the tested genes and endogenous control gene b-glucuronidase (GUSB) using the primer and probe sequences commercially (Applied Biosystems).

### Western blot analysis

Whole protein was extracted by M-PER Mammalian Protein Extraction Reagent (Thermo Scientific) from cell lines added with Phosphatase Inhibitor Cocktail Set II (Calbiochem, San Diego, CA, USA) and Complete Protease Inhibitor Cocktails (Roche, Lewes, UK) according to manufactures' protocols. The proteins were separated on 4–15% gradient SDS–polyacrylamide gels and transferred to Immobilon-P membranes (Millipore, Bellerica, MA, USA). The following primary antibodies were used: anti-CK2α (Millipore), anti-Notch1 (Cell Signalling), anti-Hes1 (BD Biosciences, San Jose, CA, USA) and anti-GAPDH (Trevigen, Gaithersburg, MD, USA). After being incubated with appropriate secondary antibodies, the antigen-antibody complexes were detected by using an ECL blotting analysis system (Amersham Pharmacia Biotech, Piscataway, NJ, USA). Digital images were prepared using Adobe Photoshop 6.0.

### Protein degradation assay

The CK2α- and control siRNA-transtected A549 cells were exposed to 50 μg/ml cycloheximide and harvested at the time-points of 0 and 1 and 2 hrs. Total cellular proteins were extracted and were analysed by western blot analysis.

### Luciferase reporter assays

To measure Notch1 transcriptional activity, the luciferase reporter constructs, 8 × wild-type Notch binding site (8 × CBF1^wt^ Luc) or 8 × mutant Notch binding site (8 × CBF1^mut^ Luc) plasmids (provided by Dr. Diane Hayward, Baltimore, MD, USA) 22, and a human Notch1 expression vector ICN1 (intracellular domain of the Notch receptor, Addgene, Cambridge, MA, USA), were cotransfected into A549 cells in 24-well plates. The Renilla luciferase pRL-TK plasmid (Promega, Madison, WI, USA), whose expression is driven by the housekeeping thymidine kinase gene promoter, was cotransfected to normalize for transfection efficiency. All transfection experiments were performed using the Lipofectamine2000 (Invitrogen) in accordance with the manufacturer's instructions. After 24 hrs cells were lysed and luciferase assays were performed as described previously 23. Results are expressed as fold induction, which is the ratio of luciferase activity induced in ICN1-transfected cells relative to basal luciferase activity in control transfected A549 cells. All experiments were performed in triplicate; means and standard errors were calculated using Student's *t*-test.

### Flow cytometry analysis

CD44^+^/CD24^−^ cells were identified as described previously. Cells were washed and then trypsinized into single cell suspensions. Combinations of fluorochrome-conjugated monoclonal antibodies obtained from BD Biosciences against human CD44 (FITC, 555478) and CD24 (PE, 555428) were added to the cell suspension at concentrations recommended by the manufacturer and incubated at 4°C in the dark for 30–40 min. Labelled cells were washed in the wash buffer to eliminate unbound antibody, and then analysed no longer than 1 hr after staining on a BD Accuri C6 flow cytometer.

### Statistical analysis

Data were expressed as mean ± S.D. from three independent experiments. All of the statistical analyses were performed using SPSS 13.0 for Windows software system (SPSS Inc., Chicago, IL, USA). Student's *t*-test was used to compare the differences among groups. A significant difference was declared if the *P* value from a two-tailed test was <0.05 (*P < 0.05, **P < 0.01).

## Results

### Notch1 signalling is activated in NSCLC

**Fig. 1:**
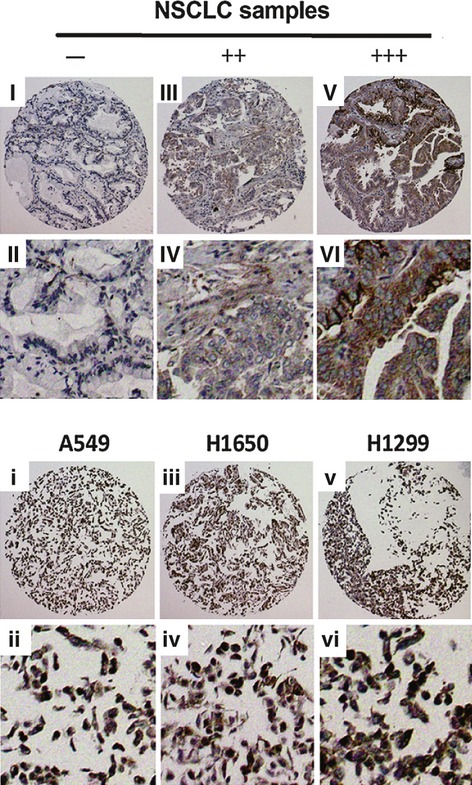
Notch1 Genes Are Over-Expressed in Human non–small-cell lung cancer (NSCLC). Immunohistochemistry Notch1 staining of NSCLC samples and cell lines. NSCLC samples (I−VIII). (I, II) −, (III, IV) ++, (V, VI) +++; (i, ii) A549, positive; (iii, iv) H1650, positive; (v, vi) H1299, positive. Images were taken with a 20× magnification.

Notch1 are over-expressed in a variety of cancers, including lung cancer. Through the use of quantitative RT-PCR (Figure S1), we examined the Notch1 expression in eight of the NSCLC lines (A549, A427, H460, H1299, H1650, H358, H838 and H322). All of the eight cell lines over-express Notch1 at the mRNA level. Normal lung was used as a negative control. The positive or negative results of Notch1 staining in NSCLC samples and cell lines of the microarray sections are shown in Figure 1, Table 1, and Table S1. The overall positive ratio of Notch1 in NSCLC samples was 85.3%. The moderate and strong positive (++/+++) ratio was 65.8%. In the three NSCLC cell lines tested (A549, H1650 and H1299), Notch1 showed positive staining. These data suggest that Notch1 signalling is frequently overexpressed in human NSCLC. To avoid the possibility that serum may cause the overexpression of Notch1 protein, we did immunohistostaining for three additional cell lines under the same serum and culture media supplement, and we found that the different NSCLC cell lines express dramatically different levels of Notch1 protein (Figure S2). H157 cell line expressed minimal level of Notch1 protein, SW900 cell line expressed medium level of Notch1 protein, and H520 cell line expressed high level of Notch1 protein.

**Table 1:** Positive and negative number and ratio of Notch1 in NSCLC samples

− Number (ratio)	+ Number (ratio)	++ Number (ratio)	+++ Number(ratio)	Total (ratio)
6 (14.6%)	8 (19.5%)	20 (48.8%)	7 (17.0%)	41 (100%)

### Inhibition of CK2α down-regulates Notch1 transcriptional activity leading to the inhibition of Notch1 downstream genes

**Fig. 2:**
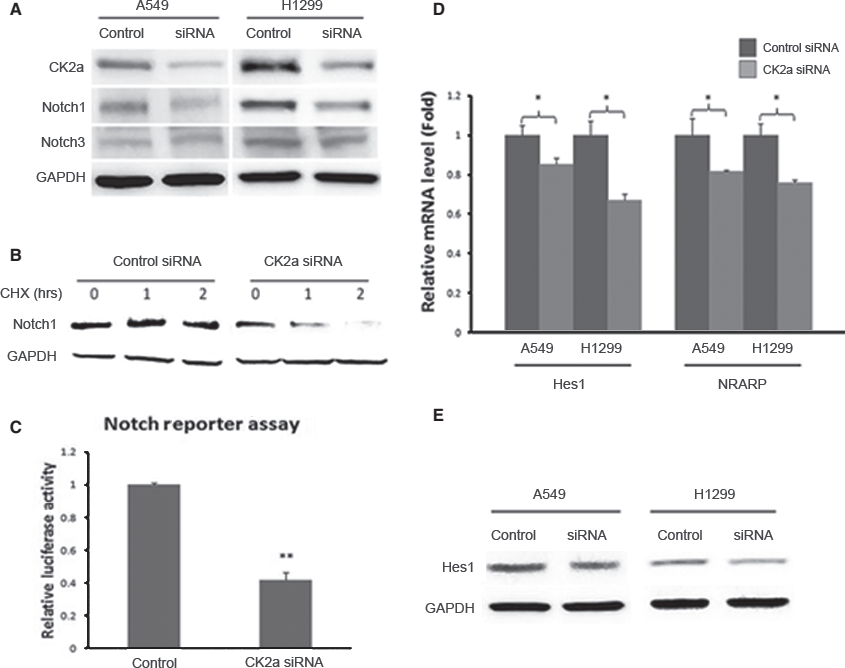
Activated Notch1 Signalling is Down-Regulated by CK2α Knockdown. (**A**) CK2α expression was silenced using siRNA knockdown. The corresponding protein level of CK2α in A549 and H1299 cell lines decreased dramatically (upper lane). The protein level of Notch1 was inhibited after CK2α knockdown both in A549 and H1299 cell lines (middle lane). GAPDH was used as a loading control (lower lane). (**B**) A time-course degradation assay was performed to examine the half-life of Notch1. In the CK2α knockdown group, the Notch1 protein level decreased dramatically from 1 hr and to minimal at 2 hrs after treatment with cycloheximide (when compared with that at 0 hr), which suggested that the half-life of Notch1 after CK2α knockdown is <2 hrs. (**C**) Luciferase reporter assay was performed to detect the transcriptional activity of Notch1 signalling. Silencing of CK2α in A549 cells resulted in a significant decrease (60% at 50 μM, *P* < 0.01) in the ICN1-boosted Notch reporter activity, compared with the non-targeting siRNA. (**D**) The mRNA level of the two Notch1 target genes decreased significantly (*P* < 0.05) after CK2α knockdown. For Hes1, the mRNA level decreased to 82% in A549 and 63% in H1299; for Nrarp, the mRNA level decreased to 80% in A549 and 74% in H1299. (**E**) At the protein level, Hes1 expression decreased after CK2α knockdown, both in A549 and H1299 cells. These data suggest a depressed transcriptional activity of the Notch1 signalling.

To investigate whether CK2 suppression has an effect on the Notch1 signalling pathway, we silenced CK2α expression using siRNA. Forty-eight hours after transfection, the efficiency of RNA interference was monitored by Western blot. The corresponding protein levels of CK2α in A549 and H1299 cell lines decreased dramatically (Fig. 2A, upper lane). The protein level of Notch1 was decreased after CK2α knockdown both in A549 and H1299 cell lines (Fig. 2A, middle lane). In addition, we performed a CK2 siRNA experiment using A427 cell line with a beta-catenin mutation. The CK2 silencing does not appear to affect the protein level of Notch1 in A427 cells (Figure S3).

Furthermore, we carried out a time-course experiment to examine the half-life of Notch1. A549 cells were transfected with CK2α or control siRNA, and Notch1 protein levels were detected at the time points of 0, 1 and 2 hrs after treatment with the protein inhibitor cycloheximide. As shown in Figure 2B, in the CK2α knockdown group, the Notch1 protein level decreased dramatically from 1 hr and to minimal at 2 hrs after treatment with cycloheximide when compared with that at 0 hr, which suggested that the half-life of Notch1 after CK2α knockdown is <2 hrs. These data indicate that CK2α knockdown results in degradation of Notch1. This, in turn, suggests that CK2α regulates Notch1 activity by preventing its degradation.

To confirm whether CK2α regulates the Notch1 signalling, we performed a luciferase reporter assay to detect the transcriptional activity of the pathway. Silencing of CK2α in A549 cells resulted in a significant decrease (60% at 50 μM, *P* < 0.01, Fig. 2C) in the Notch reporter activity, compared with the non-targeting siRNA (control).

Notch1 reportedly controls the proliferation of several cell types through various molecular mechanisms and targets downstream genes, including Hes family members and Nrarp 24. To study the effects of CK2α Knockdown on the Notch1 signalling, RT-PCR was performed to compare the levels of Hes1 and Nrarp mRNA between wild-type and CK2α knockdown cell lines. The mRNA level of the two Notch1 target genes decreased significantly (*P* < 0.05, Fig. 2D) after CK2α knockdown. For Hes1, the mRNA level decreased to 82% in A549 and 63% in H1299; for Nrarp, the mRNA level decreased to 80% in A549 and 74% in H1299. Furthermore, at the protein level, Hes1 expression decreased after CK2α knockdown, both in A549 and H1299 cells (Fig. 2E). These data suggest a depressed transcriptional activity of the Notch1 signalling.

**Fig. 3:**
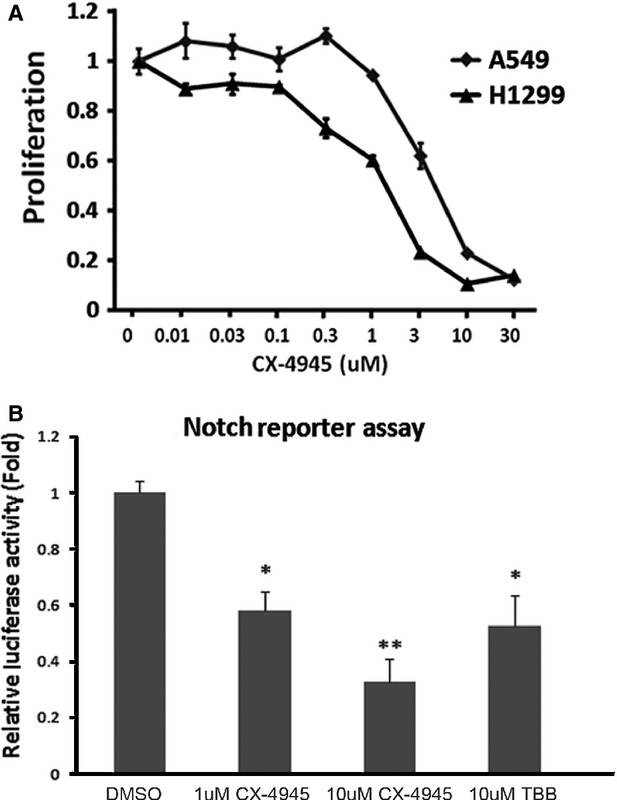
CK2 Inhibitor CX-4945 Reduces Notch1 Transcriptional Activity. (**A**) The cell proliferation assay after treatment with CX-4945. (**B**) Treatment with CX-4945 led to a dose-dependent decrease in Notch reporter activity in the A549 cell line. The decrease was 40% (*P* < 0.05) in the presence of 1 μM CX-4945 or 70% in the presence of 10 μM CX-4945 (*P* < 0.01). TBB was used as a positive control. 10 μM TBB led to a 50% decrease of Notch1 transcriptional activity.

To extend our findings to the clinical applications, we used a small-molecule CX-4945 (5-(3-chlorophenylamino)benzo[c][2,6]naphthyridine-8-carboxylic acid), a first-in-class, selective, oral inhibitor of CK2α under investigation in Phase 1 clinical trials 25. Cells were treated with multiple concentrations of CX-4945 (0.01, 0.03, 0.1, 0.3, 1, 3, 10 and 30 μM), or with the vehicle DMSO for 72 hrs. The cell proliferation assay demonstrated that treatments with CX-4945 led to cell growth inhibition in a dose-dependent manner, both in A549 and H1299 cell lines (IC50 values were 4.51 μM in A549 and 1.80 μM in H1299, respectively, Fig. 3A). We further demonstrated that treatment with CX-4945 led to a dose-dependent decrease in Notch reporter activity in A549 cell line. The decrease was 40% (*P* < 0.05) in the presence of 1 μM CX-4945 or 70% (*P* < 0.01) in the presence of 10 μM CX-4945 (Fig. 3B). TBB (4,5,6,7-tetrabromobenzotriazole), a well-known inhibitor of CK2α 26 was used as a positive control. 10 μM TBB led to a 50% decrease of Notch1 transcriptional activity.

### Overexpression of CK2α up-regulates Notch1 transcriptional activity

**Fig. 4:**
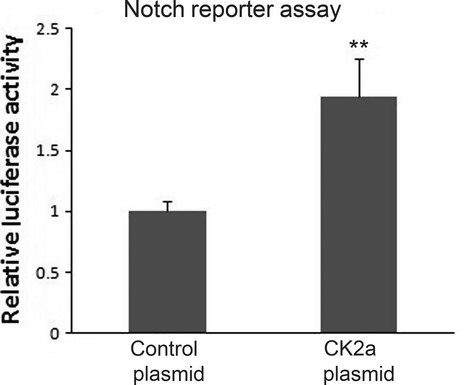
Overexpression of CK2α Activates Notch1 Transcriptional Activity. Forced overexpression of the CK2α gene was attributed to the activation of Notch1 signalling in A549 cell line. The reporter assay showed a significant (twofold, *P* < 0.01) increase of Notch1 transcriptional activity.

To further validate that CK2α positively regulates the transcriptional activity of Notch1, we transfected A549 cells with either a pcDNA3.1-CK2α or control pcDNA3.1-LacZ plasmid. As expected, forced overexpression of the CK2α gene was attributed to the activation of Notch1 signalling in A549 cell line. The reporter assay showed a significant (twofold, *P* < 0.01, Fig. 4) increase of Notch1 transcriptional activity.

### Validation of phenotypic stem-like cell reduction after CK2α inhibition

**Fig. 5:**
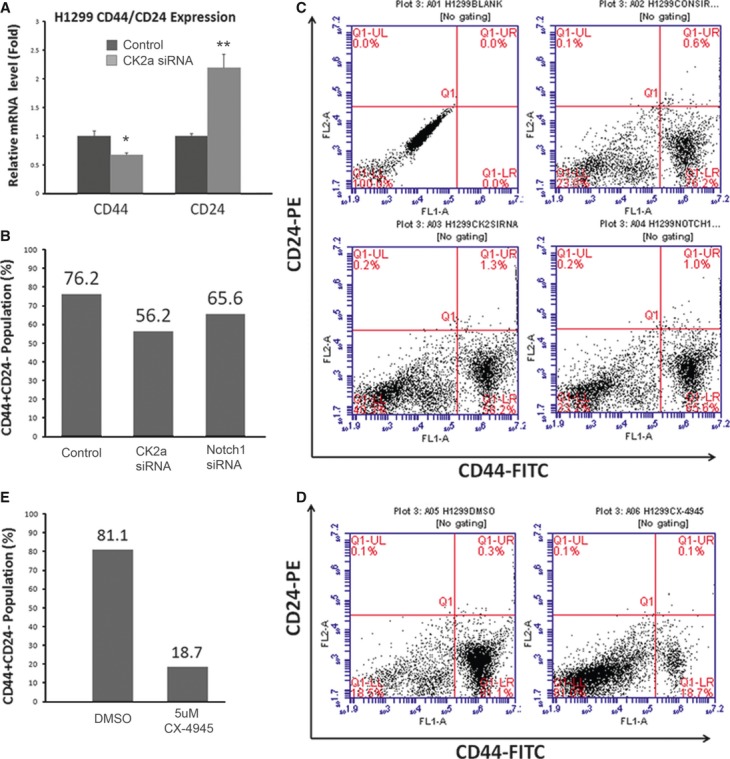
CK2 Inhibition Leads to a Reduced Proportion of CD44^+^/CD24^−^ Cells. (A) CD44 mRNA level decreased to 65% (*P* < 0.05) in CK2α-silenced H1299 cells, compared with wild-type H1299 cells (left). In analysis of CD24, CK2α-silenced H1299 cells showed relatively higher CD24 mRNA level (>twofold, *P* < 0.01; right). (B) Relatively higher percentage (76.2%) of H1299 cells were detected as CD44^+^/CD24^−^ cells. After treatment with CK2α siRNA, the proportion of CD44^+^/CD24^−^ population dropped to 56.2%. Notch1 siRNA was used as a positive control, where the proportion dropped to 65.6%. (C) Bar chart for (B). (D) After treated with 5 μM CX-4945, H1299 cells showed a strong suppression of CD44^+^/CD24^−^ proportion (from 81.1% to 18.7%). (E) Bar chart for (D).

CD44^+^/CD24^−^ phenotype has been implicated to be a stem cell marker in human cancers 27, 28. In non–small-cell lung cancer, cells expressing CD44 are enriched for stem cell-like properties. Recent studies show that CD44^+^/CD24^−^ population can be regulated by Notch1 signalling pathway which is involved in stem cell maintenance 29. Here, to study the effects of CK2α on cancer stem cell maintenance *via* regulating Notch1, we first examined CD44 and CD24 expression after treatment with 50 μM CK2α siRNA. We found that CD44 mRNA level decreased to 65% (*P* < 0.05) in CK2α-silenced H1299 cells, compared with wild-type H1299 cells (Fig. 5A, left). In analysis of CD24, CK2α-silenced H1299 cells showed relatively higher CD24 mRNA level (>twofold, *P* < 0.01; Fig. 5A, right). In further flow cytometry analysis, relatively higher percentage (76.2%) of H1299 cells was detected as CD44^+^/CD24^−^ cells in our study. After treatment with CK2α siRNA (50 μM), the proportion of CD44^+^/CD24^−^ population dropped to 56.2%. Notch1 siRNA (50 μM) was used as a positive control, where the proportion dropped to 65.6% (Fig. 5B and C). In brief, we showed a 35.6% reduction of CD44^+^/CD24^−^ proportion after CK2α knockdown.

We also analysed the proportion of CD44^+^/CD24^−^ population after treatment with CK2 inhibitor CX-4945. A strong suppression, from 81.1% to 18.7%, was showed after treatment with 5 μM CX-4945 (Fig. 5D and E).

## Discussion

Our results suggest that CK2 is a positive regulator in Notch1 signalling in human lung cancer. Several lines of evidence support this. First, the inhibition of CK2α by siRNA or small-molecular inhibitors resulted in down-regulation of Notch1 protein level and transcriptional activity. Second, forced overexpression of CK2α resulted in increased Notch1 transcriptional activity. Third, several Notch1 signalling target genes (Hes1, Nrarp and CD44) were down-regulated after CK2α knockdown. Finally, CK2α knockdown led to a reduction of the CD44^+^/CD24^−^ subtype, a stem cell-like population.

Notch signalling has a critical role in regulating cell-to-cell communication during embryogenesis, cellular proliferation, differentiation and apoptosis 30. Clinical studies indicate that 30% of NSCLC cases have increased Notch1 activity and 10% of lung squamous cell carcinomas have gain-of-function mutation of the Notch1 gene 19, 31. Notch1 also stimulates survival of lung adenocarcinoma cells 32. In this study, our data suggest that Notch1 signalling is frequently overexpressed in human NSCLC, including stage I lung adenocarcinomas (Table S1). Notch3 has also been implicated to play oncogenic role in lung cancer; however, the specific role for Notch2 or Notch4 in lung cancer is less clear 33. To date, two clinical trials have involved the secretase inhibitor RO4929097. The first one is a maintenance study for non-progressing advanced NSCLC patients after first-time therapy, with biological correlates planned that includes evaluation of Notch expression and microRNA levels. The second study is a phase I/II in combination with erlotinib in advanced-stage NSCLC 30, 34.

To date, there is no evidence that CK2 is a positive regulator of Notch1 signalling. To investigate the potential mechanism is through which CK2 regulates Notch1 signalling, we performed a protein degradation assay of Notch1 after treatment with CK2α siRNA. Our data (Fig. 2B) showed that CK2α silencing reduces the half-life of human Notch1 protein in A549 cells, suggesting that this positive regulation is partially because of increased Notch1 stability by CK2α. In addition, we found two predicted CK2 phosphorylation sites in human Notch1 by using Scansite with medium stringency (Figure S4) 35. Recently, it was reported that Notch can be phosphorylated by CK2 at serine 1901 and this phosphorylation negatively regulates Notch transcriptional activity 36. Possibly, the phosphorylation of second serine site (S847) of Notch1 by CK2 increases the stability of Notch1 protein and thus leads to increased Notch1 transcriptional activity.

As CK2 is involved in key self-renewal pathways such as Hedgehog/Gli1 and Wnt, it is also possible that CK2 positively regulates Notch1 signalling through other mechanisms, such as pathway cross-talk 37. For instance, CK2 could potentially promote Notch ligand JAG2 expression through Gli1 signalling 16, 37, and therefore its inhibition may lead to the down-regulation of JAG2/Notch1 expression. Furthermore, CK2 could also potentially stimulate the expression of JAG1 through canonical Wnt signalling, and its inhibition may lead to reduced Notch1 transcriptional activity 37. In a previous study, we showed that the correlation between the CK2α expression and Gli1 expression 16, and we also found a similar correlation between CK2α expression and Notch1 expression (data not shown). Therefore, it may be possible that CK2α also regulates Notch1 mRNA expression through pathway cross-talk. Further studies are warranted to elucidate the precise mechanisms.

The NSCLC cell lines A549 (K-Ras mutation/p53 wild-type/CDKN2A null) and H1299 (K-ras wild-type/p53 null/CDKN2A null) are two of the most widely studied NSCLC cell lines. The p53 status does not appear to be relevant for the CK2α effect on Notch1 or Notch3 in NSCLC cells (Fig. 2). Interestingly, H1299 cells without wild-type p53 appear to be more sensitive to the CK2α inhibitor CX-4945 than A549 cell with wild-type p53 (Fig. 3A). This suggests that CK2 inhibitors may induce apoptosis in these cells through a p53-independent mechanism. The data from A427 cell line (K-Ras mutation/p53 wild-type/CDKN2A null) with a beta-catenin mutation indicate that CK2 silencing does not appear to affect the protein level of Notch1 in A427 cells (Figure S3). This suggests that cancer cell lines with a beta-catenin mutation may respond to CK2 inhibitor through a different mechanism or pathway. More studies are warranted to dissect CK2 inhibition effect in lung cancer cells.

A subpopulation of CD44^+^/CD24^−^ cells has been reported to have stem/progenitor cell properties. Notch1 inhibition alters the CD44^+^/CD24^−^ population and reduces the formation of brain metastases from breast cancer 20. In NSCLC cell line H1299, cells expressing CD44 are enriched for stem cell-like properties 38. For instance, it was demonstrated that the CD44^+^ H1299 lung cancer cells survive and form spheres *in vitro* while the CD44^−^ H1299 cells do not 38. In addition, the CD44^+^ H1299 cells are also tumour initiating cells in a xenograft model 38. In our study, we found that CD44 mRNA level decreased significantly in CK2α-silenced H1299 cells, while in analysis of CD24, CK2α-silenced H1299 cells showed relatively higher CD24 mRNA level (Fig. 5A). As expected, the CD44^+^/CD24^−^ population was reduced in CK2α-silenced as well as CX-4945 treated H1299 cells. Thus, we report that CK2 participates in cancer stem cell maintenance by regulating Notch1 signalling. Interestingly, treatment with 5 μM CX-4945 led to a relatively stronger CD44^+^/CD24^−^ suppression (Fig. 4D and E). This may be partially because of the down-regulation of other self-renewal pathways by the CK2 inhibitor.

Although Notch1 signalling may play key roles in the maintenance of cancer stem cells, the druggable targets in Notch1 signalling are limited. CK2 provides an additional target for inhibition of Notch1 signalling. However, CK2 inhibitors have not been extensively developed as therapeutic agents, partly because the ATP-binding pocket of CK2 is not as druggable as some other protein kinases. To date, only one small-molecule CK2 inhibitor has been tested in clinical trials as a potential anticancer drug. CX-4945, a highly selective CK2 small molecule inhibitor, is a promising first-in-class oral therapeutic agent targeting multiple human cancers. CX-4945 shows a favourable safety profile in Phase I clinical trials 39. In addition, CIGB-300 (a synthetic peptide-based drug targeting the CK2 phosphoaceptor domain) has proved to be safe and of clinical benefit in Phase I cervical cancer trials 40, 41.

In summary, we report that CK2α is a positive regulator in the Notch1 signalling pathway, and that inhibition of CK2α down-regulates Notch1 signalling in human lung cancer cells. Given the emerging importance of Notch1 signalling in tumour initiation and progression, our findings provide important evidence for the potential benefits of CK2 inhibitors.

## Supporting information

**Figure S1.** Quantitative RT-PCR for Notch1 expression in the eight cell lines. Overexpression of Notch1 was detected in all of the eight lung cancer cell lines at the mRNA level. Normal lung was used as a negative control.

**Figure S2.** Immunohistochemistry Notch1 staining of three additional NSCLC cell lines. Three different NSCLC cell lines express dramatically different levels of Notch1 protein. H157 cell line expressed minimal level of Notch1 protein, SW900 cell line expressed medium level of Notch1 protein, and H520 NSCLC cell line expressed high level of Notch1 protein. Images were taken with a 40 9 magnification.

**Figure S3.** Effect of Notch1 by CK2α siRNA in A427 cells. CK2α expression was silenced using siRNA knockdown in A427 cell line. The corresponding protein level of CK2α decreased dramatically (upper lane). The protein level of Notch1 was not affected after CK2α knockdown in A427 cells with an activating β–catenin mutation (middle lane). Endogenous GAPDH was used as a loading control (lower lane).

**Figure S4.** The Phosphorylation Prediction Results of Human Notch1 with CK2. Two CK2 phosphorylation sites in Notch1 were predicted using Scansite 2.0 with medium stringency.

**Table S1.** Study population and specimens. Expression of Notch1 was detected in 41 NSCLC tumours using immunohistochemistry. Intensity of Notch1 staining was scored from low (−) to high (+++). Information of study population was collected: pathology, stages of tumour, ages of the patients at diagnosis and smoking history of the patients. X, the information is unknown.
