# Supplementary figures and images for: Inhibition of CK2α down-regulates Notch1 signalling in lung cancer cells
Source: J Cell Mol Med. 2013 May 8;17(7):854–62. doi: 10.1111/jcmm.12068 (PMC3729857; doi:10.1111/jcmm.12068)

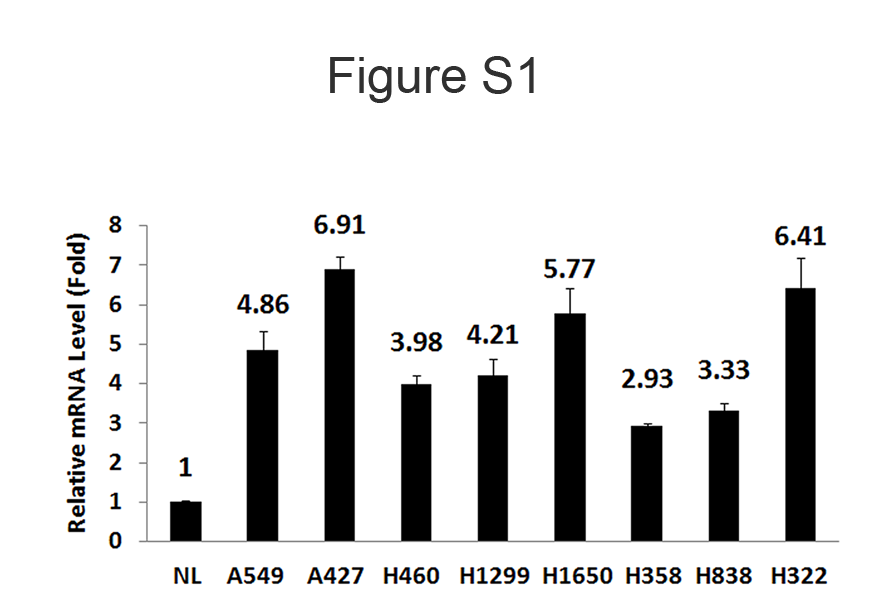

Supplement: Supplementary file 1 [file jcmm0017-0854-SD1.tif]

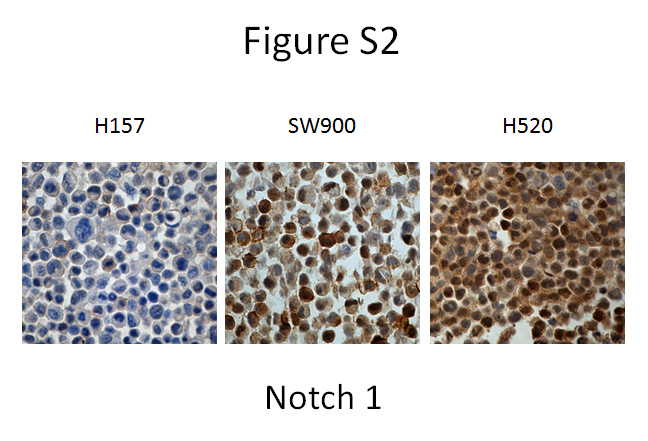

Supplement: Supplementary file 2 [file jcmm0017-0854-SD2.tif]

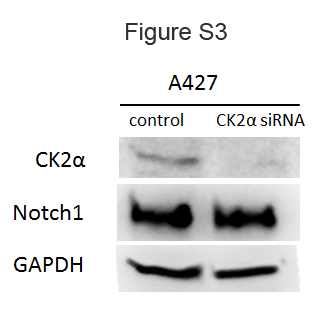

Supplement: Supplementary file 3 [file jcmm0017-0854-SD3.tif]

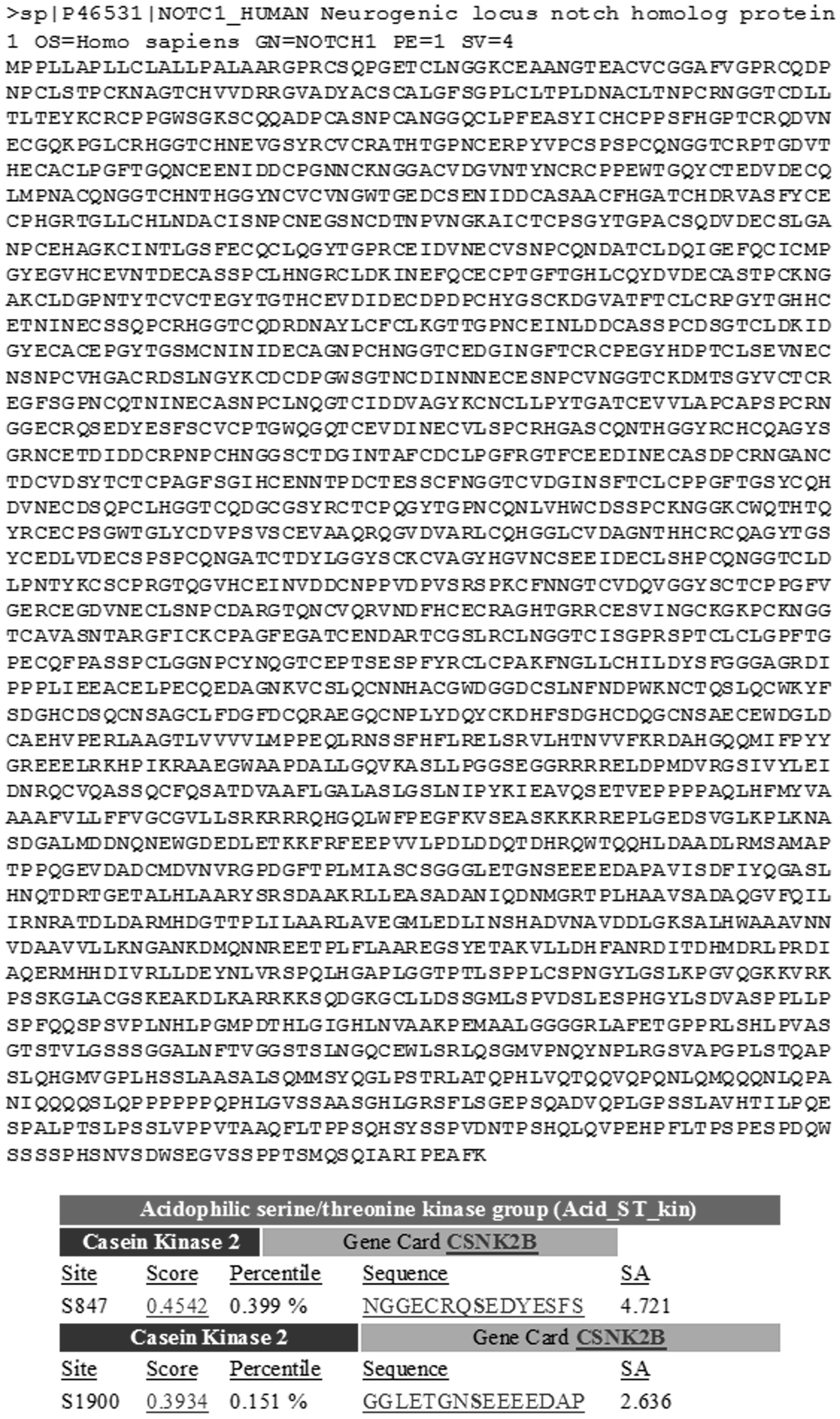

Supplement: Supplementary file 4 [file jcmm0017-0854-SD4.tif]
