# Supplementary material for: Inhibition of CK2α down-regulates Notch1 signalling in lung cancer cells
Source: J Cell Mol Med. 2013 May 8;17(7):854–62. doi: 10.1111/jcmm.12068 (PMC3729857; doi:10.1111/jcmm.12068)
Supplement: Supplementary file 5 [file jcmm0017-0854-SD5.docx]

| **Sample number** | **IHC of**  **anti-Notch1** | **Pathology** | **Age**  **at diagnosis** | **Stage** | **Smoking history** |
| --- | --- | --- | --- | --- | --- |
| T1 | ++ | ADENOCARCINOMA | 63 | X | X |
| T2 | ++ | ADENOCARCINOMA | 64 | X | X |
| T3 | +++ | ADENOCARCINOMA | 64 | X | X |
| T4 | - | ADENOCARCINOMA | 69 | X | X |
| T5 | + | ADENOCARCINOMA | 48 | X | X |
| T6 | ++ | ADENOCARCINOMA | 74 | II | X |
| T7 | +++ | ADENOCARCINOMA | 58 | I | X |
| T8 | + | ADENOCARCINOMA | 79 | X | X |
| T9 | + | ADENOCARCINOMA | 75 | X | X |
| T10 | ++ | ADENOCARCINOMA | 67 | I | X |
| T11 | +++ | ADENOCARCINOMA | 69 | X | X |
| T12 | - | ADENOCARCINOMA | 73 | I | X |
| T13 | ++ | ADENOCARCINOMA | 61 | IIIA | X |
| T14 | + | ADENOCARCINOMA | 68 | II | X |
| T15 | ++ | ADENOCARCINOMA | 75 | I | X |
| T16 | - | ADENOCARCINOMA | 70 | II | X |
| T17 | + | ADENOCARCINOMA | 72 | IIIA | X |
| T18 | - | ADENOCARCINOMA | 67 | I | X |
| T19 | + | ADENOCARCINOMA | 45 | IIA | X |
| T20 | + | ADENOCARCINOMA | 66 | I | X |
| T21 | ++ | SQUAMOUS CELL CARCINOMA | 70 | X | X |
| T22 | ++ | ADENOCARCINOMA | 74 | IA | X |
| T23 | +++ | ADENOCARCINOMA | 83 | I | X |
| T24 | ++ | ADENOCARCINOMA | 71 | II | Smoker |
| T25 | ++ | ADENOCARCINOMA | 68 | I | X |
| T26 | ++ | ADENOCARCINOMA | 73 | IIIA | X |
| T27 | ++ | ADENOCARCINOMA | 73 | IIIB | X |
| T28 | ++ | ADENOCARCINOMA | 61 | II | X |
| T29 | ++ | ADENOCARCINOMA | 79 | II | X |
| T30 | ++ | ADENOCARCINOMA | 82 | I | Smoker |
| T31 | - | ADENOCARCINOMA | 44 | X | X |
| T32 | +++ | ADENOCARCINOMA | 67 | I | X |
| T33 | ++ | ADENOCARCINOMA | 59 | X | X |
| T34 | + | ADENOCARCINOMA | 48 | III | X |
| T35 | - | ADENOCARCINOMA | 63 | IV | X |
| T36 | ++ | ADENOCARCINOMA | 62 | X | X |
| T37 | ++ | ADENOCARCINOMA | 52 | IV | X |
| T38 | +++ | ADENOCARCINOMA | 47 | X | Non-smoker |
| T39 | ++ | ADENOCARCINOMA | 69 | X | X |
| T40 | ++ | ADENOCARCINOMA | 62 | IIB | X |
| T41 | +++ | ADENOCARCINOMA | 63 | I | X |
